# Vaping among Norwegians who smoke or formerly smoked: reasons, patterns of use, and smoking cessation activity

**DOI:** 10.1186/s12954-023-00768-z

**Published:** 2023-03-21

**Authors:** Ingeborg Lund, Gunnar Sæbø

**Affiliations:** Department for Alcohol, Tobacco and Drugs, Norwegian Institute of Public Health (NIPH), Folkehelseinstituttet, Postboks 222, 0213 Skøyen, Oslo Norway

**Keywords:** e-Cigarettes, Vaping, Smoking cessation, Adults

## Abstract

**Background:**

The majority of Norwegians who use e-cigarettes are adults who have smoked. Little is known about vaping reasons and -patterns in this group. The aim of this paper was to study vaping prevalence, patterns, and motivations among adults who smoke. Furthermore, to investigate smoking intensity and smoking cessation behaviour differences between those who vape and those who do not.

**Methods:**

This study was based on two separate Norwegian samples: People who had ever smoked, from 2017 (*N* = 2099), and people who currently smoked and recent quitters, from 2018/2019 (*N* = 1336). Measures of vape frequencies, vape motives, and smoking cessation behaviours were utilised in descriptive analyses of relationships between vaping and smoking behaviour.

**Results:**

Less than 1 in 10 in the ever-smoked group, 1 in 5 of the currently smoked or recently quit group, were currently vaping. Ever trial rates for vaping were much higher at 1 in 3 in the ever-smoked group, and 1 in 2 in the currently smoked or recently quit group. Dual use with combustible cigarettes was common, but people who smoked tended to use e-cigarettes less frequently while those who formerly smoked tended to use them more frequently. Both quitting attempts and smoking intensity reduction were positively associated with vaping, and the most common reasons for e-cigarette use were reported to be desires to reduce harm, to stop smoking, or to reduce smoking intensity.

**Conclusion:**

The results indicate that Norwegians who smoke tend to see e-cigarettes as a tool to reduce or completely stop smoking. The predominance of use-motivations related to reducing harm points at the importance of conveying correct information about relative harmfulness of tobacco- and nicotine products.

## Background

After more than 60 years of anti-tobacco regulations, smoking remains a serious public health problem, causing large losses in healthy years of life, and contributing to maintaining and possibly increasing, social inequality in health [1–3]. Consequently, best practice in the prevention of smoking is crucial to public and individual health. This also includes a need to understand the complexity of contemporary nicotine and tobacco landscapes, particularly the implications following from differences in harmfulness between various tobacco and nicotine products. In the presence of this harm diversity, regulatory authorities confront the challenge of achieving a balance between two opposing objectives: To shelter the young from nicotine addiction and the harmful effect of combustible tobacco and simultaneously, to minimize the tobacco-related burden of disease among adults who smoke [4, 5].

While the protection of the young favours a strict approach to new nicotine and tobacco products, a more lenient approach might arguably be better suited for reducing harm in the smoking population [6]. Specifically, to retain accessibility of less harmful products means providing reduced-risk alternatives to adults who smoke to use as smoking cessation aids, or as replacement products in the event that abstinence is not achieved. Furthermore, as preferences, tastes, and nicotine addiction levels will vary between people who smoke, an array of reduced harm options might stimulate smoking cessation and product substitution in more individuals than one would achieve in a situation with fewer product choices.

The e-cigarette, also known as a vaporizer, is a relative newcomer to the tobacco and nicotine market. Recent evidence suggests that although e-cigarette use (vaping) can cause acute physical reactions associated with respiratory and cardiovascular distress [7], and even though nicotine itself may have some harmful effects, particularly salient for adolescents and pregnant women [8], vaping likely involves substantially less short-term risk to health than smoking [6]. Evidence on long-term health effects is limited [9, 10]. Since they were launched in 2006 [11], e-cigarettes have become popular as replacement products among people who smoke worldwide, and results from two Cochrane reviews [9, 12] suggest that e-cigarettes with nicotine might help people to stop smoking. Furthermore, some evidence suggest that vaping increases the likelihood of smoking reduction among people who continue to smoke [12]. Common internationally is a pattern of people both vaping and smoking [13], but it is not known if vaping might delay smoking cessation or increase the risk of relapse to smoking among people who formerly smoked who have maintained their nicotine addiction [14]. A pattern of both vaping and smoking does not seem to increase adverse events and undesirable changes in biomarkers compared to exclusive cigarette smoking [15, 16]. However, research also suggest that using both products will not necessarily lead to any reductions in these biomarkers [17].

In Norway, vaping is subject to the same restrictions as smoking, including a ban on indoor use (Directorate of Health, 2017). Vaping can therefore not be used as a replacement product in places where smoking is banned. Additionally, as the Norwegian Tobacco Act of 1989 prohibits entry of any new nicotine or tobacco product on the domestic market, inland sale of nicotine-containing e-liquids is banned. Domestic shops selling devices and no-nicotine liquids exist, but the number of physical outlets is relatively low. Despite these barriers, approximately 150.000 people in the country currently vape [18]. According to existing information from representative surveys (age 16+), the average age of Norwegian people who vape is 43 years, and the vast majority (97%) of regular vapers are currently or have formerly been smoking [19]. At present, Norwegian vapers import 80 per cent of their e-liquid and 60 per cent of their vaporizers from retailers abroad and over the internet [19]. However, the planned ratification of the tobacco product directive (TPD) in 2023 will likely change these percentages, as nicotine-containing e-liquid for the first time will be legal to sell in domestic outlets. This significant shift in the e-cigarette’s legal status might change who vapes and the prevalence of vaping [20]. With the deregulation in the wake of TPD as a backdrop, knowledge on how e-cigarettes might be rendered useful in smoking cessation is important.

No study has investigated the e-cigarette’s effect on smoking prevalence in Norway. However, studies on snus, a lower harm smokeless tobacco with long traditions of use in Scandinavia, support a reduction in smoking prevalence when less harmful nicotine-containing products are available [21, 22]. A deeper recognition of how people who smoke use e-cigarettes, and the reasons they have for using them, might help us understand some of the mechanics at work in contemporary tobacco and nicotine markets, and improve our knowledge about how to make the most of the e-cigarettes’ potential smoking-reducing effect.

In this study, we address reasons for, and patterns of, e-cigarette use among Norwegian adults who smoke or formerly smoked. Furthermore, we investigate if people who use e-cigarette differ from people who don’t in terms of smoking cessation activities, and discuss the occurrence of dual use.

## Methods

This study was based on two separate samples of people who currently smoke or formerly smoked. A summary of the most central differences between the samples is reported in Table 1. Importantly, while both samples included both people who currently or formerly smoked, the time of smoking cessation is not known, and could potentially be anytime in Sample 1, but was restricted to have happened during the last 1–1½ years in Sample 2.

**Table 1:** Description of materials and measurements for smoking and vaping

	Sample 1	Sample 2
Data collection	Autumn 2017	Autumn 2018
Smoking history	Smoking and former smoking with unknown quit date	Smoking and former smoking with quit date within the last 1–1½years
Age (mean)	15–90 (45.7)	19–84 (54.6)
Prp women	49%	54%
*N*	2099	1336
Smoking measurement	Daily, occasional, former daily	Daily, occasional, quit after spring 2017 (wave 1)
Vaping measurement	Weekly or more often, less than weekly, have only tried once or a few times, never tried	Daily, weekly, monthly, less than monthly, tried earlier, never tried

### Sampling

#### Sample 1

Data were collected during the period November 8—December 18, 2017, as part of a nationwide survey on tobacco habits and public support for novel tobacco control policies. The sample was randomly drawn from the online panel of the commercial pollster NORSTAT, stratified by gender, age, region, and education to be representative of the entire population and consists of 4002 people between the ages of 15 and 90. Informed consent was obtained from all subjects involved in the study, and the study was approved by the Data Protection Officer at the Norwegian Institute of Public Health.

Of these 4002 respondents, 10.1% (*n* = 406) smoked daily, 10.4% (*n* = 415) smoked occasionally, while 31.9 (*n* = 1278) formerly smoked. All respondents who had ever smoked constitute the body of material 1 (*N* = 2 099).

#### Sample 2

Data were collected in the autumn/winter of 2018/2019, as the second wave of a 2-wave online survey on tobacco habits and tobacco-pack related opinions among Norwegian adults who smoke (*N* = 1336, 19–84 years, mean = 54.6, 54% women). Participants were recruited from the commercial pollster Kantar’s online panel, using current tobacco smoking as inclusion criterion. Kantar also carried out the collection of data.

All participants gave written consent to participate, and the Data Protection Officer at the Norwegian Institute of Public Health approved the study. The sample includes 1336 respondents, out of which 1050 individuals had participated also in wave 1 (spring 2017), while 288 individuals were recruited at wave 2.

While all participants smoked at recruitment, some of the wave-1 recruits had quit smoking in the time gap between waves, and the sample therefore contains both people who smoke and people who formerly smoked, with the distribution of smoking habits being: 63.5% (*n* = 848) daily smoking, 19.3% (n = 258) occasional smoking, and 17,7% (*n* = 230) former smoking. All the 288 wave-2 recruits smoked daily or occasionally. Further details about the sampling procedure are found in Lund and Lund [23].

### Measures

In both materials, the **proportion of vapers** was measured by the question “Have you ever tried an e-cigarette?” (yes/no), while f**requency of vaping** was measured by the follow-up “How often do you use e-cigarettes now?” In Sample 1, the frequency question had response alternatives “weekly of more often” (*regular use*), “less than weekly” (*occasional use*), and “have only tried once or a few times” (have *tried*). In Sample 2, response alternatives daily, weekly, monthly, less than monthly, or not at all, were transformed to *regular use* (daily or weekly), *occasional use* (monthly or less than monthly), and have *tried* (ever using and reporting not at all on current use) to increase comparability between the two materials. For logistic regressions, bivariate alternatives were constructed such that occasional and regular use were considered vaping (1), while never use and experimenting were considered no vaping (0).

**Fig. 1:**
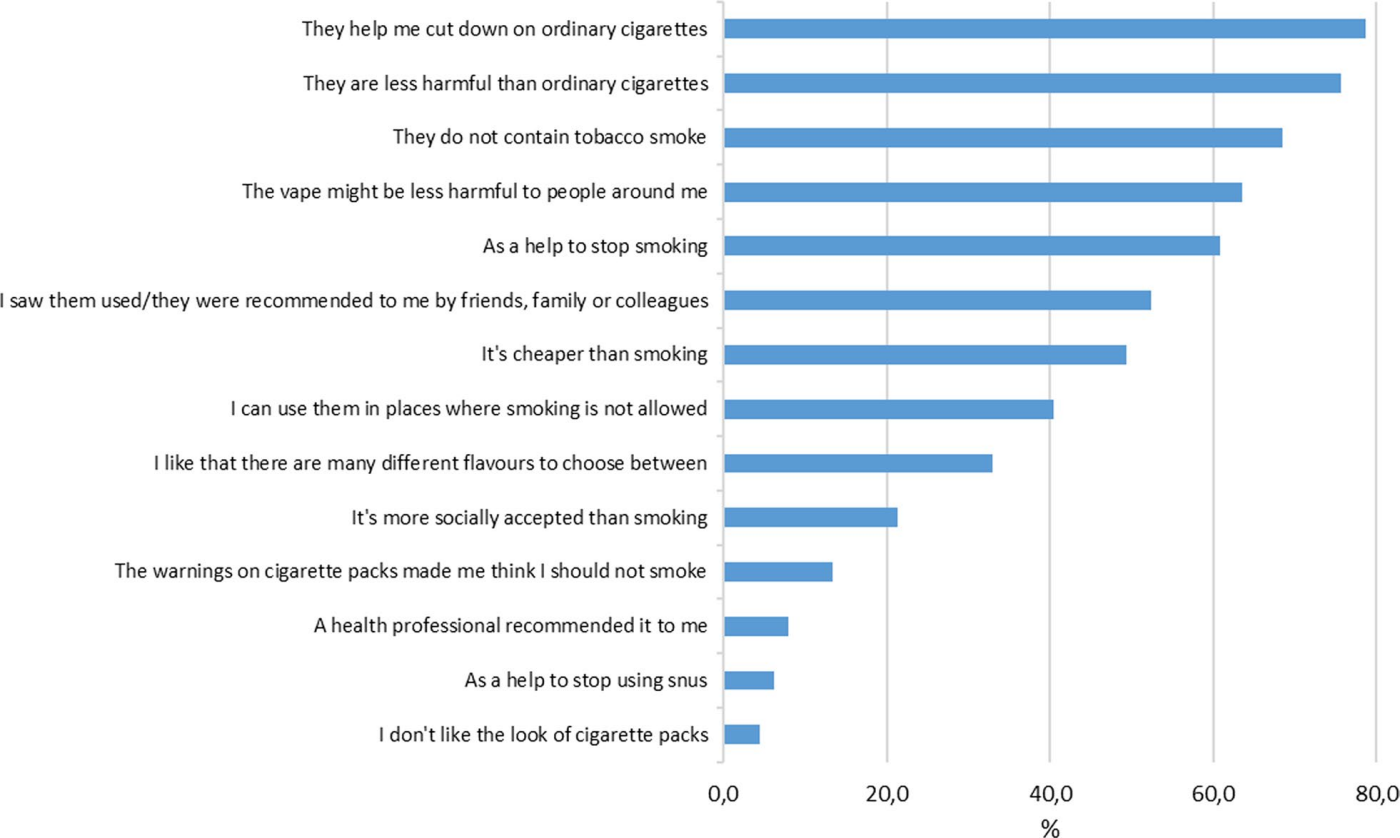
Reasons for use of e-cigarettes as cited by current vapers (Sample 2, multiple choice, *N* = 225)

In Sample 2, all regular and occasional vapers were also asked to indicate by answering yes or no, which ones of 14 given statements that best described their **reasons for vaping** (“which of these reasons best describe your decision to try e-cigarettes?”, see Fig. 1 for the complete list of given statements).

In Sample 1, information on **smoking status** was retrieved from the question “What are your current smoking habits?” (I smoke daily, I smoke occasionally, I do not smoke), with the additional question to those who did not smoke daily: “Have you previously smoked daily” (yes/no). In Sample 2, smoking status was measured by the question “Which alternative best suits your situation?” with response alternatives “I smoke cigarettes every day”, “I smoke cigarettes but not every day”, “I have quit smoking”. In both materials, people who smoked reported last 12 months **quitting attempts** (yes/no), and indicated any **quitting plans** within the next 6 months (yes/no). Furthermore, Sample 2 included a question on current **smoking reduction** attempts (yes/no). Sample 1 included a measure of intention to **not be smoking daily in five years,** where those who answered ‘will certainly not smoke on a daily basis’ were contrasted with all other response categories collapsed (‘I will definitely smoke on a daily basis’, ‘am likely to smoke on a daily basis’, ‘will probably not smoke on a daily basis’).

### Statistical analyses

Descriptive bivariate analyses and logistic regression analyses were applied. In the bivariate analyses, crosstabs and chi-square tests were used to estimate statistical significance. Pairwise deletion of missing data was used in the bivariate analyses of Sample 2, while in sample 1 there was no missing data, as the web questionnaire was designed to force answers on all items. All analyses were performed using the statistical software package SPSS 27.

## Results

### Vaping and smoking frequencies

69.7% of Sample 1, and 50.2% of Sample 2 had never tried e-cigarettes, while 23.4% and 32.5%, respectively, had tried but were not current vapers (Table 2). The proportion of current vapers (occasional + regular) was lower in Sample 1 (6.8%) than in Sample 2 (17.3%). There was a significant association between smoking status and e-cigarette trial and use in both samples, with more trial, occasional and regular use among people who smoked daily than among those who smoked occasionally. In Sample 1, ever-use of e-cigarettes was less common among those who had quit smoking than among people who smoked, while this was not the case in Sample 2 where people who had quit smoking had tried e-cigarettes to the same extent as those who occasionally smoked.

**Table 2:** Use of e-cigarettes (%) within smoking categories in Sample 1 (*N* = 2099) and Sample 2 (*N* = 1300)

	Former smoking	Occasional smoking	Daily smoking	Total
*Sample 1 (Ever-smoked)*				
Never tried	84.1	51.8	42.9	69.7
Have tried	12.8	38.3	41.6	23.4
Occasional vaper	0.6	5.1	9.1	3.1
Regular vaper	2.4	4.8	6.4	3.7
(*N*)	(1278)	(415)	(406)	(2099)
*Sample 2 (Smoked or recently quit)*				
Never tried	55.7	59.8	45.8	50.2
Have tried	30.4	25.6	35.2	32.5
Occasional vaper	3.5	8.9	11.8	9.8
Regular vaper	10.4	5.7	7.3	7.5
(*N*)	(230)	(246)	(824)	(1300)

Chi sq = 0.001 in both materials

In both samples, people who formerly smoked and were currently vaping were more likely to be regular vapers, while people who continued to smoke more often were occasional vapers. Calculations based on the figures in Table 2 show for example that in Sample 2, 22% of ever vapers in the daily smoking group were current occasional vapers, while 13% of them were regular vapers (calculating percentages within the group of 447 people in the daily smoking group who had ever tried or used e-cigarettes). In comparison, in the group that formerly smoked, the corresponding proportions were 8% occasional, and 24% regular vapers. Similar tendencies were seen in Sample 1.

Dual use of e-cigarette and ordinary cigarettes was common in both samples. In Sample 1, 10% in the occasional smoking group and 15.5% in the daily smoking group were also current vapers. Similarly, in Sample 2, current vaping was reported by 14.6% of those who smoked occasionally, and 19.1% of those who smoked daily.

### Reasons for vaping

When given a list of potential reasons for using e-cigarettes, the most commonly endorsed statements were those associated with smoking reduction, reduced health risks for oneself or others, and smoking cessation (Fig. 1). Almost 4 in 5 current vapers (78.7%) reported that a reason for vaping was that e-cigarettes made it easier to reduce smoking, while approximately 3 in 4 (75.6%) reported the lower harm to health from e-cigarettes compared to regular cigarettes as a reason. The lack of tobacco smoke in e-cigarette vape (68.4%), the potential for reducing harm to others (63.6%), and the use of e-cigarettes as a smoking cessation tool (60.9%) were also reasons that achieved high scores.

On the lower end of the scale, statements concerning the social acceptability of smoking (21.3%), worries caused by health warnings on cigarette packs (13.3%), and the look of cigarette packs (4.4%), had low scores. Very few had used e-cigarettes to quit snus (6.2%), and less than one in ten (8.0%) had tried vaping because a health professional had recommended it. Additional analyses (not reported in table) showed only minor differences in justifications for use among women and men, and between age groups.

### Smoking cessation behaviour

Among vapers, the highest proportion of cigarette quitters was seen within the group of regular vapers (40% and 30% in Sample 1 and 2, respectively, while the lowest proportions were seen within the group of occasional vapers, at circa 12% and 9% (Table 3). In Sample 1, never-vapers had more often than others quit smoking (73%). This was however not the case in Sample 2, where the proportion of (last 1 ½ year) cigarette quitters among never-vapers (25%) lay between the proportion among vape triers and regular vapers.

**Table 3:** Cessation activities within vaping categories. Both samples

	Vaping					Chi sq
	Never tried	Not current	Occasional	Regular	Total	
*Sample 1 (Ever smoked)*						
Have quit*	73.4	33.3	12.1	40.3	60.9	0.000
Last year quit attempt**	21.1	29.6	39.7	47.8	27.3	0.000
6 month quit plan**	49.1	49.7	58.6	67.4	51.0	0.069
No daily smoking in 5 years**	46.0	41.2	24.1	32.6	41.8	0.008
*N	1464	492	66	77	2099	
**N	389	328	58	46	821	
*Sample 2 (Current smoking and recent quitting)*						
Last year quit	25.0	21.8	8.5	30.4	22.9	0.002
Last year quit attempt	31.4	37.9	40.7	46.9	35.6	0.006
6 month quit plan	37.2	37.1	41.6	48.4	38.5	0.329
Trying to reduce smoking	53.5	55.5	64.4	72.6	56.7	0.005
N***	435–633	307–428	101–128	62–98	905–1300	

*All respondents who smoke or formerly smoked. **Respondents who still smoke. ***Variation in Ns due to item non-response

Last year quit attempts were reported significantly more often by vapers than by non-vapers, with the highest proportions of quit attempts seen among regular vapers. Similar tendencies were seen for 6 months quitting plans, although these were not statistically significant. People who smoked daily and were occasional vapers were significantly less likely to think they would have quit daily smoking in 5 years, compared to people who smoked daily and were non-vapers or regular vapers. There was also a significant association between vaping and current attempts at reducing smoking intensity, reported by 73% of regular vapers and 54% of never-vapers.

In logistic regression analyses, measuring vaping binary (never or only tried vs. occasional or regular use), sample 1 participants who smoked occasionally (AOR = 2.56, *p* < 0.001) or daily (AOR = 5.69, *p* < 0.001) were significantly more likely to use e-cigarettes than people who had quit smoking. In sample 2 people who smoked daily (AOR = 1.85, *p* < 0.01) were significantly more likely to use e-cigarettes, while there was no significant difference between people who smoked occasionally and those who had quit smoking during the last year in this respect.

Additional adjusted logistic regressions (not reported in table) were carried out with vaping as the dependent variable and quit behaviours among people who currently smoked as independent variables (simultaneously adjusting for last year quit attempt, 6-month quit plan, smoking frequency, age, and gender, for both samples, in addition to no daily smoking in 5 years for sample 1, and trying to reduce smoking for sample 2). Results showed significant associations in sample 1 (*N* = 821) between vaping and last year quit attempt (AOR = 1.98, *p* < 0.01) and planning not to smoke daily in 5 years (AOR = 0.49, *p* < 0.01). In sample 2 (*N* = 652), a significant association was found between vaping and currently trying to reduce smoking (AOR = 1.78, *p* < 0.05). In both samples there was also a significant association between age and vaping, with effect sizes similar to the ones in Table 4.

**Table 4:** Adjusted associations between vaping and smoking status

	Sample 1 (*N* = 2099)		Sample 2 (*N* = 1045)	
	AOR	95% CI for AOR	AOR	95% CI for AOR
Smoking (ref = quit/quit last year)				
Occasionally	2.56***	1.57–4.17	1.09	0.62–1.93
Daily	5.69***	3.74–8.66	1.85**	1.21–2.83
Women	0.78	0.55–1.11	0.88	0.63–1.93
Age	0.98***	0.97–0.99	0.99	0.97–1.00

Logistic regression adjusting for all independent variables simultaneously. Dep. variable: never or only tried vaping (0) versus occasional or regular use (1)

*AOR* Adjusted odds ratio, *CI* confidence interval

***p < 0.001, **p < 0.01

## Discussion

In this two-sample study, less than 1 in 10 who had ever smoked were currently using e-cigarettes, with the proportion doubling to almost 1 in 5 in a sample where all had been smoking 1½ year earlier. Ever trial rates for vaping were at much higher levels, ranging between 1 in 3, and 1 in 2 in our two samples. Dual use with combustible cigarettes was common, but people who currently smoked tended to vape less frequently than people who formerly smoked. Among recent cigarette quitters (Sample 2), 24% of ever-vapers vaped weekly or daily, while the corresponding proportion in the daily smoking group were 13%. Positive associations were established between vaping frequency and attempts at quitting or reducing smoking, and the most common reasons for e-cigarette use were reported to be desires to reduce harm, to stop smoking, or to reduce smoking intensity.

While Norwegian adults who smoke have good knowledge of the harms to health from smoking, results suggest that they are less well informed about the risk profile of e-cigarettes [24], and particularly that they often underestimate the degree to which e-cigarettes are less harmful [25]. However, the predominance of vaping motivations related to harm reduction and smoking cessation in this study, indicates that at least those who use vaporizers are aware of a marked difference in the harm potential of the two products. Furthermore, these motivations, which also correspond to vaping motivations reported in other European countries [26], support the notion that people who smoke are interested in reducing the risk of health damage due to nicotine delivery. This highlights the importance not only of the availability of less harmful nicotine products, but also of making available correct information on relative risks to give people who smoke the best possible foundation to take rational action [5].

The higher occurrence of attempts at smoking cessation and reduction found among vapers is in line with earlier Norwegian results [27] and harmonizes with the reported motivations for vaping. Furthermore, a high prevalence of daily and weekly vaping among ever-vapers who formerly smoked suggests that e-cigarettes may have worked well for some individuals and have enabled them to achieve their goal of reduced harm.

While the e-cigarette has not been conceptualized or promoted as a smoking cessation tool by the health authorities, recent results suggest that people who use them in combination with cessation counselling will have equal success at quitting smoking as people using NRTs under similar circumstances [28, 29]. However, health professionals tend not to recommend e-cigarettes for smoking cessation, often due to being sceptical about their efficiency or being worried about the harmfulness of nicotine [30–32]. Furthermore, to have been suggested vaping by a health professional was one of the least common reasons for e-cigarette use in our sample, indicating perhaps the potential for increased cessation activity if this course of action was exploited more efficiently.

Quitting attempts were associated with vaping, but no such association could be established for quitting plans. In addition, occasional vapers more than others thought they would still be smoking daily in 5 years. A possible explanation of these seemingly incongruent findings is that vaping might play a role in spontaneous quitting. This is a factor that potentially could be of significant value, as recent international results indicate that almost half of cigarette quitters quit spontaneously [33]. Furthermore, there is some evidence suggesting that spontaneous quit attempts might be more successful in terms of achieving cigarette abstinence, than planned quit attempts [33, 34].

A noteworthy result is that the majority of the people who experimented with vaping had not continued to stable e-cigarette use. While short-term experimenting with vaping can result from curiosity, and not necessarily involve plans for long-term use, it is probable that for a sizeable proportion of these experimenters, harm reduction motivations similar to those seen for the current vapers would have been present. A timely question is, therefore, what reasons they could have had for discontinuing the vaping. Research have pointed at some e-cigarette-specific obstacles that might dissuade people who smoke from progressing from experimenting to stable use, including initial physical discomfort, e.g., coughing, or device problems like leaking, and difficulties finding a satisfactory device or flavor [35]. Furthermore, one might speculate that experimenters sometimes use e-liquids with too little, or no, nicotine, potentially due to erroneous ideas of the extent of the harmfulness of nicotine [36]. It could also be, that differences in puff frequency between vaping and smoking, and the fact that a vaping session has no clear start and finish, contrary to the act of smoking a cigarette, might have discouraged some experimenters. Finally, people who smoke might be unconvinced about vaping’s capability to deliver on aspects of smoking that are not related to nicotine uptake. As discussed in the literature, people who smoke may be addicted not just to nicotine, but to the cigarette itself [37] e.g., because they use the cigarette as a tool to communicate identity or to create a sense of community [38]. One might speculate that these obstacles could be overcome more easily if vaping was supported also by smoking cessation counseling.

The distribution of vaping across smoking categories found in this study corresponds with findings both internationally [39, 40] and in Nordic countries [41]. Notably, the large proportions of people who currently smoke among the vapers in our samples mirror international findings showing a high prevalence of dual use with ordinary cigarettes among vapers [13]. Even though this did not necessarily imply more use, it could possibly suggest prolonged smoking for some people due to sustained nicotine addiction from vaping. The higher proportion of people who formerly smoked among never-vapers than ever-vapers in sample 1 could potentially be understood to support this interpretation. However, it is important to bear in mind that many of these individuals probably quit smoking before vaporizers became available, and that a similar difference between never- and ever-vapers did not materialize in sample 2, where quitting was restrained to a timeframe when vaporizers were available. On the other hand, and in correspondence with findings from systematic reviews supporting a higher likelihood of smoking reduction among vapers [12, 42], bivariate results supported a positive association between vaping frequency and smoking intensity reduction efforts.

Given the absence of e-cigarette promotion, the ban of inland sale of nicotine e-liquid, and the fact that vaping in public places is as restricted as smoking, one might argue that vaping is surprisingly widespread in Norway, with around 150,000 current users nationally [18]. However, compared to countries with a less restrictive approach, the Norwegian prevalence of vaping is low and likely to increase after legalisation through the TPD [20]. While an increase in e-cigarette use among people who smoke may be favourable from a public health perspective (to the extent that it increases smoking reduction, quit attempts and spontaneous quitting), increased vaping among adolescents in the wake of legalisation is not welcomed by the health authorities. The regulatory challenge is thus to facilitate for e-cigarette use among adults who wants to quit (or reduce) smoking, while simultaneously hampering use among young people who do not use tobacco. To better achieve this balance, the regulatory bodies may adopt additional targeted measures to curb use among youngsters, for example advertising bans and improved enforcement of age limits.

### Limitations

The results from these analyses stem from cross-sectional data and causality can therefore not be examined. In the interpretation of results, it is also important to be conscious of the fact that the data stems from two separate samples, with significant differences in the composition of the former smoking groups. In Sample 2, all quitted smoking at a time when vaporizers were available, while in Sample 1, an unknown proportion quit smoking at an earlier time point. This probably contributes to explain the much higher proportion of never-vapers in the group who formerly smoked in Sample 1 than in Sample 2 (cf. Table 3). Furthermore, the classification of people who formerly smoked differs between samples. While in Sample 2 the group includes both those who formerly smoked occasionally and those who formerly smoked daily, only those who formerly smoked daily were included in Sample 1. The classification of current smoking, however, was similar in the two samples.

A factor that would have affected that proportion of dual users in Sample 2 is that 288 individuals recruited at the second wave by default had to be smoking at the time of survey. However, separate analyses (not reported in table) showed that the percentages of occasional and regular vaping among people who smoked daily and occasionally in this subgroup was similar to the percentages found for the total sample. Finally, the vapers were not asked explicitly about the nicotine content of the liquids they used. Even if it is likely that most respondents vape liquids with nicotine, some may also have used e-liquids without nicotine. The nicotine content in e-liquids is likely to be a factor that might affect both vaping frequency, dual use, and discontinuation of vaping among people who smoke.

### Conclusion

Contemporary nicotine and tobacco landscapes are complex and contain a duality springing from the differences in harmfulness from different products. In this cross-sectional study, we were not able to explicitly examine the causal effects of e-cigarette use on reducing the number of cigarettes or on smoking cessation. To address such questions, studies with longitudinal designs are needed. Still, the findings of the present study indicate that the e-cigarette has found a place as a tool for quitting or reducing combustible cigarette smoking in Norway. There is a need for further studies delving deeper into motivations and explanations for vaping among adults who smoke or have previously smoked. Particularly, increasing our knowledge of factors associated with dual use, and understanding better the underlying reasons for the discontinuation of vaping, might be useful in developing strategies that could help people who smoke overcome the initial difficulties in switching from cigarettes to vaporizers. In addition, further investigation into the various sub-segments of vapers, including the role of e-cigarettes among Norwegian youth, is important to complement our study of smoking cessation among adults.

## Data Availability

The datasets used and/or analysed during the current study are available from the corresponding author on reasonable request.
